# Editorial: Recent advances in biomass conversion to sustainable chemicals and polymers

**DOI:** 10.3389/fchem.2024.1475162

**Published:** 2024-08-28

**Authors:** Haixin Guo, Yan Cao, Hu Li, Jinjia Xu, Huie Zhu

**Affiliations:** ^1^ Agro-Environmental Protection Institute, Ministry of Agriculture and Rural Affairs, Tianjin, China; ^2^ Institute of Polymer Optoelectronic Materials and Devices, State Key Laboratory of Luminescent Materials and Devices, South China University of Technology, Guangzhou, China; ^3^ State Key Laboratory of Green Pesticide, Key Laboratory of Green Pesticide & Agricultural Bioengineering, Ministry of Education, State-Local Joint Laboratory for Comprehensive Utilization of Biomass, Center for R&D of Fine Chemicals, Guizhou University, Guiyang, China; ^4^ Department of Chemistry and Biochemistry, University of Missouri–St. Louis, St. Louis, MO, United States; ^5^ Graduate School of Engineering, Tohoku University, Sendai, Japan

**Keywords:** biomass, green chemistry, furan derivatives, polymers, biomass-derived fuels, catalytic mechanism

Given the increasing global concerns regarding the energy crisis and environmental pollution, renewable lignocellulosic biomass characterized by low cost and widespread availability has attracted attention as a sustainable feedstock for the production of valuable chemicals and biofuels. Despite their potential as alternatives to petroleum-based products, the recalcitrant and crystalline structures of renewable biopolymers, with their polysaccharide and lignin units, pose accessibility challenges to reagents and catalysts. Green and highly efficient processes are therefore necessary to reduce the cost of biomass-derived products for practical applications. The Research Topic “*Recent Advances in Biomass Conversion to Sustainable Chemicals and Polymers*” highlights the necessary functional materials, green conversion methods, and removal processes, with particular attention to key advancements, challenges, and future directions in this crucial field. With regard to waste valorization, the main aim here is to spotlight recent research articles and reviews dedicated to the catalytic upgradation of renewable and waste resources.

Plant-mediated synthesis approaches provide eco-friendly alternatives to conventional synthesis methods. The article “*Plant-mediated synthesis of flower-like Cu*_*2*_ *O microbeads from Artemisia campestris L. extract for catalyzed synthesis of 1,4-disubstituted 1,2,3-triazole derivatives*” by Abdelbaki et al. introduces the synthesis method for flower-like Cu_2_O microbeads from Artemisia campestris L extract and their application as catalysts. The aqueous extract was shown to effectively reduce and stabilize the Cu_2_O particles during their formation, resulting in a closely packed cubic crystal structure. The reaction parameters were optimized to explore the catalytic activities in the syntheses of 1,4-disubstituted 1,2,3-triazole derivatives, with the highest yield of 85.3% obtained in dimethylformamide. The increased yield and recyclability of the Cu_2_O microbeads highlight their potential for sustainable chemical transformations.

The paper “*Exploring the potential of* Ephedra alata *leaf extract: phytochemical analysis, antioxidant activity, antibacterial properties, and green synthesis of ZnO nanoparticles for photocatalytic degradation of methylene blue*” by Zaater et al. examines the phytochemical composition, antioxidant properties, and antibacterial activities of the *Ephedra alata* leaf extract and its application in the green synthesis of ZnO nanoparticles for the photocatalytic degradation of methylene blue. The leaf extract is shown to contain phenolic acids with specific total phenolic and flavonoid compositions. The synthesized ZnO nanoparticles have spherical shapes and are shown to degrade 87% of the methylene blue content under UV light within 90 min (at pH 7 and 25°C). This research underscores the antioxidant and antibacterial properties of the *Ephedra alata* leaf extract and its role in the green synthesis of ZnO nanoparticles that can be used in sustainable water treatment and pollution control efforts.

Ibrahim et al. show that optimized membrane composition enables high efficiency of dye removal; in their article “*Fabrication of cellulose nanocrystals/carboxymethyl cellulose/zeolite membranes for methylene blue dye removal: understanding the factors, adsorption kinetics, and thermodynamic isotherms*”, the authors report the fabrication and application of nanocellulose-based membranes to remove methylene blue dye from wastewater. The membranes are composed of 60% sugarcane-bagasse-derived cellulose nanocrystals, 15% carboxymethyl cellulose, 20% zeolite, and 5% citric acid; they are reportedly able to remove 79.9% of the dye and have an adsorption capacity of 38.3 mg/g at pH 7. The adsorption mechanisms follow pseudo-second-order kinetics and the Freundlich isotherm model. The authors successfully developed these membranes for efficient dye removal, contributing to sustainable water treatment.

In addition to renewable plant extracts, industrial wastes can be used as cost-effective sources of sustainable catalysts, as reported by El-Bayoumy et al. in their article “*Utilization of iron filing solid wastes for optimum biodiesel production*”. In this work, solid wastes composed of iron filings from mechanical workshops are used as the catalyst for biodiesel production from waste cooking oil. The iron filings as solid wastes are characterized based on their composition and particle size using various methods, such as X-ray fluorescence and particle size distribution analysis. The proposed catalyst achieves 96.4% conversion efficiency with potential for reusability. This work advocates the use of industrial solid wastes as catalysts, thereby offering a sustainable approach for energy production.

In the review paper titled “*Advances in mixed-matrix membranes for biorefining of biogas from anaerobic digestion*” by Guerrero Piña et al., the authors discuss the current status and technologies of polymeric mixed-matrix membranes (MMMs) for CO_2_/CH_4_ separation. The review focuses on the performances of these membranes in terms of permeability, selectivity, and ability to operate under low-pressure conditions that are typical in biogas systems. The review shows that intrinsic microporous polymers can be potentially used in biomethane purification owing to their properties at low pressures. The combination of these polymers and graphene oxide in the MMMs contributes to membrane stability. The authors show that incorporation of advanced nanomaterials as fillers in the MMMs promises improved gas separation performance; they also stress upon the need for further research to overcome the current limitations and achieve widespread implementation.

